# The Response of Circulating Leptin Levels to Exercise Stress Testing in Subjects Diagnosed with Metabolic Syndrome

**DOI:** 10.1155/2014/689260

**Published:** 2014-01-28

**Authors:** Dana Pop, Alexandra Dădârlat, Gyorgy Bodizs, Liana Stanca, Dumitru Zdrenghea

**Affiliations:** ^1^University of Medicine and Pharmacy, 4 Pasteur Street, Cluj-Napoca, Romania; ^2^Rehabilitation Hospital, 46-50 Viilor Street, 400437 Cluj-Napoca, Romania; ^3^Faculty of Economics and Business Administration, “Babes-Bolyai” University, Strada Teodor Mihali, No. 58-60, 400591 Cluj-Napoca, Romania

## Abstract

*Aim*. To assess the plasma leptin responses after exercise stress testing in patients with metabolic syndrome (MS). *Material and Methods*. We investigated 67 patients with MS, with mean age of 55 ± 7 years. They underwent exercise stress testing on cycloergometer. The lot was divided into three groups: group 1—10 patients with a true positive test, group 2—18 patients with a true negative test, and group 3—39 patients with a false negative test. Leptin levels were measured using the ELISA method. *Results*. Leptin levels decreased after effort in patients with MS (9.42 ± 11.08 ng/mL before and 8.18 ± 11.5 ng/mL after the exercise stress test, *P* = 0.0005, *r* = 0.874). In groups 1 (8.98 ± 9.09 at rest versus 5.98 ± 8.73 ng/mL after the exercise test, *P* = 0.002) and 3 (8.6 ± 10.53 at rest versus 6.91 ± 9.07 ng/mL, *P* = 0.0005), lower leptin levels were recorded immediately after exercise testing. Leptin levels were not significantly lower in group 2 before effort (9.49 ± 11.36 ng/ml) and after (9.46 ± 13.81 ng/mL). We found no correlation between leptinemia and exercise stress testing parameters, regardless of group. *Conclusion*. Our research showed that short-term exercise lowers leptin levels in coronary patients, without a relationship between its parameters and leptin values.

## 1. Introduction 

Lately, the adipose tissue was found to be a major endocrine organ which secretes adipokines and cytokines, called adipocytokines. Recent studies have revealed the significant role of adipocytokines in the pathogenesis of obesity, metabolic syndrome, and cardiovascular disease [1].

Thereby, studies performed on humans and animals have shown that adiponectin, apelin, or omentin exerts antiatherogenic effects through various mechanisms, while leptin, resistin, and visfatin are associated with atherosclerosis [1]. So high leptin levels are involved not only in the appearance and progression of atherosclerosis but also in the development of arterial hypertension and metabolic syndrome [1].

The adipose tissue is mainly composed of adipocytes, which produce and release active biopeptides, known as adipokines or adipocytokines. Undoubtedly, adiponectin and leptin are the most widely studied adipokines. Leptin and other proteins, secreted by fat cells or macrophages, are found in large amounts in obese patients, whereas adiponectin levels are low. Recent data shows that adipokines and adipose tissue play a substantial role in the development of obesity, metabolic syndrome, cardiovascular disease, and atherosclerosis process [2–4]. Although the role of adipokines in the pathogenesis of cardiovascular disease is not yet fully understood, there is sufficient evidence that those could be important markers of this pathology [2, 3].

In this context, the present study aims to assess the way in which leptin levels are influenced by exercise stress testing in patients with or without myocardial ischaemia diagnosed with metabolic syndrome.

## 2. Materials and Methods

The study was performed on 67 patients diagnosed with metabolic syndrome, who were admitted in the Cardiology Department of the Rehabilitation Hospital, Cluj-Napoca, Romania. The selected patients were informed about the study protocol and gave their signed informed consent. The Institutional Ethics Committee of “Iuliu Hatieganu” University of Medicine and Pharmacy approved the study protocol. The mean age was 55 ± 7 years. 42 (62.68%) were female patients and 25 (37.32%) were men. Metabolic syndrome was defined using the International Diabetes Federation (IDF) criteria [1]. All patients were assessed for the presence of cardiovascular risk factors (obesity, arterial hypertension (HTN), fasting plasma glucose levels, metabolic syndrome, smoking, and plasma levels of LDL cholesterol and triglycerides). Blood glucose was measured using the glucose oxidase method. Serum lipids, total cholesterol, triglycerides, and high density cholesterol were measured using commercially available kits (Spinreact, Sant Esteve de Bas, Spain; performance characteristics—Table 1). Low density cholesterol was estimated using the Friedewald formula.

Blood pressure was measured as the mean of two readings after the participant was at rest for 5 minutes in a sitting position, according to the standard protocol. All patients underwent maximal symptom-limited exercise treadmill testing (ETT) using the standard Bruce protocol. Exercise capacity was measured in METs (metabolic equivalents of oxygen consumption). 1 MET is a unit of sitting/resting oxygen uptake (*≈*3.5 mL of O_2_ per kilogram of body weight per minute [mL·kg^−1^·min^−1^]).

Heart rate, blood pressure, and 12-lead ECGs were recorded before exercise, at the end of each exercise stage, and after the first minute of the recovery phase. Thus, the patients were divided into three groups: group 1 contained 10 patients (5 women) with a true positive exercise stress testing (ST segment depression induced during effort or in recovery of 1 mm or more abnormal test result in individual with disease), group 2 included 18 patients (10 women) with a true negative treadmill stress testing (no ischaemic ST changes during the exercise test or immediately after effort, reaching or exceeding 85% of maximum predicted heart rate-normal test result in individual without disease), and group 3 included 39 patients (27 women) with a false negative test (no ischaemic ST changes during the exercise test or immediately after effort, but without achieving 85% of maximum predicted heart rate-normal test result in individual with disease). In all subjects, plasma levels of leptin were measured using the ELISA method (Quantikine, R&D Systems, Abington, UK). Venous blood samples were obtained prior to ETT (sample I) and 30 min after the exercise was stopped (sample II). Statistical analysis was carried out by using the SPSS 16.0 Software for Windows (Demo Version), *P* value (Student's *t* test), and Pearson's correlation index.

## 3. Results

The characteristics of the patients are summarized in Table 2.

**Figure 1:**
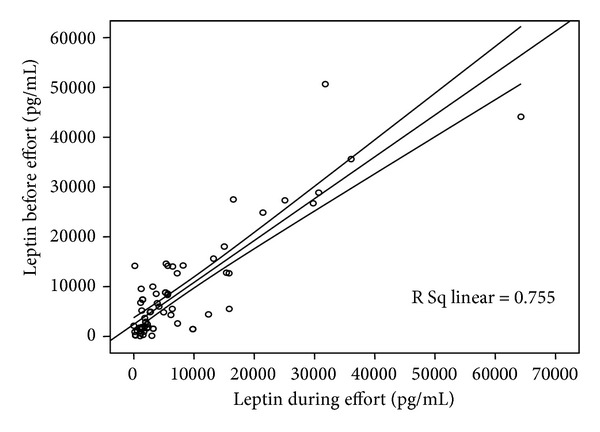
Plasma leptin levels before and immediately after effort.

Plasma leptin levels in patients with metabolic syndrome were 9.42 ± 11.08 ng/mL before performing the exercise testing. Those significantly decreased after effort, reaching values of 8.18 ± 11.5 ng/mL (*P* = 0.0005 and *r* = 0.874)—Figure 1.

The mean BMI recorded in each group was group 1: 29.75 ± 3.4, group 2: 30.75 ± 4.4, and group 3: 31.50 ± 5.2.

In both groups 1 and 3 lower levels of circulating leptin were recorded in immediate postexercise recovery (group 1: 8.98 ± 9.09 at rest versus 5.98 ± 8.73 ng/mL after the exercise test, with a *P* value of 0.002 and *r* value of 0.903, and group 2: 8.6 ± 10.53 ng/mL at rest versus 6.91 ± 9.07 ng/mL, *P* = 0.0005, *r* = 0.876).

Conversely, plasma leptin levels were not significantly different in patients from group 2 before treadmill stress testing (9.49 ± 11.36 ng/mL) and after it (9.46 ± 13.81 ng/mL, *P* > 0.05). There were no statistically significant differences between mean values of leptin before and after the treadmill stress test in none of these three groups of patients.

We found no correlation between leptin levels and exercise stress testing parameters in patients in recovery after the exercise test, regardless of group—Table 3.

## 4. Discussion

Hyperleptinemia causes increased insulin resistance [5], induction of endothelial dysfunction, stimulation of inflammatory reaction, oxidative stress, hypertrophy, and proliferation of vascular smooth muscle cells [1].

Our study showed no statistically significant differences between plasma leptin values in rest or after effort in none of the three groups, even though patients from groups 1 and 3 (with true positive or false negative treadmill stress tests) were far more likely of being diagnosed with ischaemic heart disease than those with true negative tests. True negative exercise stress tests exclude the diagnosis of ischemic heart disease.

In return, we found a statistically significant decrease in leptin levels after exercise testing in all patients with metabolic syndrome. Also, lower levels of circulating leptin were found immediately after exercise testing in patients with certain or probable diagnosis of ischaemic heart disease (groups 1 and 3). However, in group 2 there were no statistically significant decreases in plasma leptin values.

It is well known that regular moderate-intensity exercise is substantially beneficial to ischemic heart disease patients. Besides increasing exercise capacity in these patients, physical exercise also improves endothelial dysfunction and decreases certain inflammatory mediators, such as C-reactive protein [6], superoxide dismutase, 8-isoprostane, and CD34/KDR+ endothelial progenitor cell count [7]. At the same time, long-term exercise has important antiatherogenic and anti-ischaemic effects [8].

There are many studies that have investigated circulating leptin variations depending on the type of exercise performed, such as short- or long-term exercise and regular physical workout [9]. However, most of the studies, especially those in which the effort was short-lived, were performed on healthy patients. Thus, Kraemer et al. carried out a study on 15 postmenopause women, who performed treadmill stress testing at 80% VO_2_ max. They found no significant changes in leptin levels over 2 hours and 10 min baseline, whether or not women received hormone replacement therapy [10]. Similar results were reported also by Pérusse et al. [11]. At the same time, Houmard et al. showed that even though short-term exercise in healthy sedentary lean subjects does not affect leptin levels, it improves insulin sensitivity index [12].

On the other hand, several trials including healthy subjects have shown that long-term exercise, such as cycling or athletics, led to lower leptin levels immediately after exercise [13–17]. Those results could be explained by the alterations in the distribution and flow of nutrients in the adipocytes, which occur during prolonged physical exercise. The alterations interfere with the secretion of leptin from the adipocytes with a consequent decrease of its plasma levels [9]. In the present study, leptin values were not statistically significantly decreased after exercise in subjects with metabolic syndrome without ischemic heart disease (group 2). Ozcelik et al. reported no significantly decreased leptin levels immediately after exercise in obese women [18].

Unlike patients with a true negative test (group 2), patients with a true positive (group 1) or false negative test (group 3) presented with lower leptin concentrations immediately after exercise testing. These decreases in leptin values lead to several discussions. There is data substantiating leptin involvement in the development of cardiovascular events, in restenosis after angioplasty, and also in the occurrence of strokes [2, 19–22]. It seems that chemical structure of leptin is similar to that of cytokines. So leptin can stimulate the activity of monocytes and macrophages with an increasce of several inflammatory factors secretion, such as TNF-*α*, IL-6, IL-12, and Th 1 cytokines. Those inflammatory factors are involved in the development and progression of atherosclerosis process [23]. As mentioned above, besides its central role in obesity, leptin also increases insulin resistance, which is involved in the pathogenesis of both metabolic syndrome and ischemic heart disease [24–26]. Also, the ADVANCE (Atherosclerotic Disease, Vascular Function, and Genetic Epidemiology) study revealed an association between leptin and coronary calcifications in elderly women [27]. In another study called SIRCA (Study of Inherited Risk of Coronary Atherosclerosis), circulating leptin was associated with inflammatory factors (IL-6, CRP, and TNF-*α*) and also represented an independent marker for coronary artery calcifications [22].

There are questions remaining about the fact that only those patients with possible or doubtless diagnosis of ischaemic heart disease showed decreases in plasma leptin levels immediately after effort. We speculate that physical exercise lowers leptin levels in those patients due to its antioxidative, anti-inflammatory, and antiatherogenic effects. We found no statistically significant correlation between leptin levels and exercise stress testing parameters (WATT_s_, MET_s_, and heart rate during effort). We should emphasize that the average maximal heart rate was about 80%, being close to 85%, which is the submaximal heart rate set by the exercise stress testing guidelines [28].

There are no studies regarding the relationship between short-term effort and fluctuations of plasma leptin levels in patients suffering from ischaemic heart disease.

Although our study includes a small number of patients, it reveals that physical exercise of any type contributes to achieving lower leptin plasma levels. Undoubtedly, further studies including a larger number of patients are needed, in order to confirm that exercise intensity (WATT_s_ and MET_s_) does not influence leptin values.

In conclusion, short-term exercise lowers leptin levels in coronary patients, without a relationship between its parameters and plasma leptin values.

## Figures and Tables

**Table 1:** Performance characteristics of chemistry and immunologic tests used in our study.

Analite	Method	Sensitivity	Intra assay precision (CV)	Inter-assay precision (CV)
Fasting blood sugar (FBS)	GOD-PAP	1.0 mg/dL	0.83%	1.58%
Total cholesterol	CHOD-PAP	1.0 mg/dL	0.61%	3.05%
HDL cholesterol	Direct, enzymatic, and colorimetric	3 mg/dL	1.2%	0.93%
Triglycerides	GPO-PAP	1 mg/dL	0.58%	3.38%
Leptin	ELISA	7.8 pg/mL	3%	4.2%

**Table 2:** Main characteristics of the subjects.

Variables	Values
Number	67
Females (%)	62.68 (42)
Age (yr.)	55 ± 7
Fasting blood sugar (FBS) (mg/dL)	111 ± 48
Total cholesterol (mg/dL)	195 ± 57
LDL cholesterol (mg/dL)	117 ± 40
HDL cholesterol (mg/dL)	45 ± 14
Triglycerides (mg/dL)	117 ± 40
BMI* (kg/m^2^)	30.66 ± 4.3
Systolic blood pressure (mmHg)	186 ± 28.16
Diastolic blood pressure (mmHg)	91.15 ± 7.79
WATT_s_	103.81 ± 29.29
MET_s_**	5.458 ± 1.43
Maximal heart rate in effort (beats/minutes)	128.933 ± 21.97
Heart rate (beats/minutes)	78.271 ± 11.85
Leptin at rest (ng/mL)	9.42 ± 11.08
Leptin after effort stress testing (ng/mL)	8.18 ± 11.5

*Body mass index; **1 MET: 1 metabolic equivalent (MET) is a unit of sitting/resting oxygen uptake (*≈*3.5 mL of O_2_ per kilogram of body weight per minute [mL·kg^−1^·min^−1^]); maximal heart rate for men = 220 − age (years); maximal heart rate for women = 210 − age (years).

**Table 3:** The relationship between exercise stress testing parameters and circulating leptin values in recovery phase of treadmill stress test.

Leptin levels in recovery phase of treadmill stress test	WATT_s_ (mL·kg^−1^·min^−1^)	MET_s_	Heart rate during effort	Maximal heart rate
All patients				
Pearson's correlation index	−0.062	−0.031	0.191	0.155
*P* value	0.642	0.812	0.144	0.237
Group 1				
Pearson's correlation index	0.210	0.086	0.487	0.482
*P* value	0.651	0.854	0.268	0.273
Group 2				
Pearson's correlation index	−0.0146	−0.131	0.89	0.033
*P* value	0.396	0.447	0.606	0.848
Group 3				
Pearson's correlation index	−0.009	−0.129	0.061	-0.062
*P* value	0.968	0.547	0.777	0.773
